# JAK2V617F‐dependent down regulation of SHP‐1 expression participates in the selection of myeloproliferative neoplasm cells in the presence of TGF‐β

**DOI:** 10.1111/jcmm.70138

**Published:** 2024-10-21

**Authors:** Céline Aoun, Nabih Maslah, Saravanan Ganesan, Norman Salomao, Romane Gendron, Sarah Awan Toor, Gil Letort, Panhong Gou, Mélina Bonnamy, Véronique Parietti, Jean‐Jacques Kiladjian, Stephane Giraudier, Bruno Cassinat

**Affiliations:** ^1^ Inserm UMRS_1131, Institut de Recherche Saint‐Louis, Université de Paris Paris France; ^2^ Laboratoire de Biologie Cellulaire, APHP Hopital Saint‐Louis Paris France; ^3^ Université Paris Cité, INSERM/CNRS, US53/UAR2030, Institut de Recherche Saint‐Louis Paris France; ^4^ Centre d'Investigations Cliniques, APHP Hopital Saint‐Louis Paris France

**Keywords:** JAK2, Myeloproliferative neoplasms, SHP‐1, TGF‐β

## Abstract

Myeloproliferative neoplasms (MPNs) are characterized by an increased production of blood cells due to the acquisition of mutations such as JAK2^V617F^. TGF‐β, whose secretion is increased in MPN patients, is known to negatively regulate haematopoietic stem cell (HSC) proliferation. Using an isogenic JAK2^V617F^ or JAK2 wild‐type UT‐7 cell line we observed that JAK2^V617F^ cells resist to TGF‐β antiproliferative activity. Although TGF‐β receptors and SMAD2/3 expressions are similar in both cell types, TGF‐β‐induced phosphorylation of SMAD2/3 is reduced in UT‐7 JAK2^V617F^ cells compared with JAK2 WT cells. We confirmed that JAK2^V617F^ mutated cells are resistant to the antiproliferative effect of TGF‐β in a competitive assay as we observed a positive selection of JAK2^V617F^ cells when exposed to TGF‐β. Using cell lines, CD34‐positive cells from MPN patients and bone marrow cells from JAK2^V617F^ knock‐in mice we identified a down regulation of the SHP‐1 phosphatase, which is required for the regulation of HSC quiescence by TGF‐β. The transduction of SHP‐1 cDNA (but not a phosphatase inactive cDNA) restores the antiproliferative effect of TGF‐β in JAK2^V617F^ mutated cells. Finally, SC‐1, a known agonist of SHP‐1, antagonized the selection of JAK2^V617F^ mutated cells in the presence of TGF‐β. In conclusion, we show a JAK2‐dependent down regulation of SHP‐1 in MPN patients' cells which is related to their resistance to the antiproliferative effect of TGF‐β. This may participate in the clonal selection of cancer cells in MPNs.

## 1 INTRODUCTION

BCR::ABL1 negative myeloproliferative neoplasms (MPNs) include polycythemia vera, essential thrombocythemia and primary myelofibrosis. These haematological malignancies are characterized by an increased proliferation of haematopoietic myeloid lineages leading to an excess of mature blood cells. One main physio‐pathologic and diagnostic parameter is the presence of disease driver mutations in the *JAK2*, *CALR* or *MPL* genes, the JAK2^V617F^ mutation being the most frequent mutation in all MPN subtypes.^1^ These mutations, acquired at the level of haematopoietic stem and progenitor cells (HSPCs),^2^, ^3^, ^4^ all lead to a constitutive activation of the JAK2/STAT5 pathway. The JAK2^V617F^ mutation, which abolishes the JH2‐mediated negative regulation of the JAK2 protein activation, leads to a cytokine‐independent constitutive activation of the kinase activity of JAK2 and hence, to the deregulated proliferation of HSPCs.^5^ MPNs are clonal disorders with progressive accumulation of JAK2^V617F^‐mutated cells, which is attributed to an increased fitness of the mutant haematopoietic progenitors,^6^ a growth advantage of these progenitors in the presence of low erythropoietin (EPO) levels and to a selective advantage of JAK2^V617F^‐HSPCs in the context of the MPN microenvironment.^1^ The latter is characterized by increased levels of a large number of cytokines including inflammatory cytokines produced by the mutant cells in the bone marrow niche.^7^ Such an inflammatory micro‐environment^8^ may participate to the selection of JAK2^V617F^‐HSPCs.^9^

HSPCs are located in specialized bone marrow microenvironment, or ‘niche’ where they respond to a variety of signals emanating from soluble factors and surrounding cells. Among the cytokines secreted at high levels by JAK2‐mutated cells in the bone marrow niche is the transforming growth factor beta (TGF‐β), a member of a large family of cytokines involved in an array of cellular processes. In addition to its well‐known effect in the development of myelofibrosis,^10^, ^11^ TGF‐β is involved in the maintenance of HSPCs in a quiescent state by inhibiting the cell cycle in these cells, as shown in various models.^12^, ^13^, ^14^ Although TGF‐β production is increased in megakaryocytes from MPN patients,^15^ it does not prevent the expansion of JAK2‐mutated HSPCs, which invade the bone marrow of patients. The resistance of JAK2^V617F^ cells to TGF‐β could be due to a reduced TGF‐β signalling in MPN HSPCs (intrinsic mechanism), or the secretion of TGF‐β inhibitors by cells from the microenvironment. To understand the mechanisms of the resistance of MPN cells to TGF‐β we undertook a gene expression analysis in JAK2‐mutated cells and identified for the first time a reduced expression of the *PTPN6* gene, coding for the SH2 domain‐containing phosphatase 1 (SHP‐1), in JAK2^V617F^ cells. Because previous studies reported that SHP‐1 participates to the TGF‐β signalling^16^ we explored whether its dysregulated expression could be involved in the resistance of JAK2^V617F^‐mutated cells to the growth inhibitory activity of TGF‐β, and hence in the clonal selection of these mutant cells.

## 2 MATERIALS AND METHODS

### 2.1 Cell lines and reagents

UT‐7 JAK2 WT and JAK^2V617F^ cell lines were previously generated through lentiviral transduction of a TMJ‐GFP JAK2 WT or mutant plasmid into the human megakaryoblastic UT‐7 cell line expressing the receptor to EPO. The JAK^2V617F^ UT‐7 cells expressing m‐cherry were generated for this study through lentiviral transduction of pLV‐mCherry (Addgene©). UT‐7 sub‐clones used in this study were cultured in low glucose DMEM media (Gibco©) supplemented with 10% fetal bovine serum (FBS), 1% Penicillin–Streptomycin and EPO (1 IU/mL). The JAK2^V617F^ mutated human erythroblastic HEL cells were obtained from the American type culture collection and cultured in RPMI 1640 medium supplemented with 10% FBS and penicillin–streptomycin (Thermo Fisher Scientific©). The JAK2^V617F^ mutated human megakaryoblastic UKE‐1 cells were cultured in IMDM medium with 20% FBS, 1% of Penicillin–Streptomycin, and hydrocortisone (1 μmol/L). The cell lines were tested and determined to be free of mycoplasma. For TGF‐β treatment, cells were cultured in serum‐free StemSpan SFEM media (Stem Cell Technologies©), devoid of any TGF‐β to prevent biases. The SHP‐1 agonist SC‐1 was purchased from Sigma©. Cultured cells were maintained in a humidified atmosphere at 37°C with 5% CO2. Ruxolitinib was purchased through Selleckchem (Cat. no.: S1378).

### 2.2 Patients derived CD34 positive cells

Blood samples from JAK2^V617F^ mutated MPN patients were obtained after written informed consent from Saint‐Louis Hospital in Paris, France. Blood samples from healthy donors of similar ages (50–60 years old) were provided by EFS (Etablissement Français du Sang). After the isolation of peripheral blood mononuclear cells on a ficoll gradient (Eurobio‐scientific©), CD34 positive cells were isolated using the EasySep™ Human CD34 Positive Selection Kit II (Stemcell Technologies©) following manufacturer's instructions.

### 2.3 Western blotting

The antibodies used for western blotting are indicated in Table S1. The primary antibodies were diluted 1/1000 in T‐PBS + 5% BSA or, in the case of STAT3, T‐PBS + 5% non‐fat dry milk. Following primary antibody incubation, membranes were washed in T‐PBS before being incubated with the appropriate secondary antibodies for 1 h at room temperature. The secondary antibodies were diluted 1/10.000 in T‐PBS + 5% non‐fat dry milk. After additional washing in T‐PBS, the membranes were incubated for 1 min with ECL ‘pico’ (Cat. no.: 34580, Thermo Scientific) or Super Signal ‘Femto’ (Cat. no.: 34596, Thermo Scientific) and bands were revealed using the Amersham Image Quant 800. Quantification and analysis were performed using the Image Lab software.

### 2.4 Quantitative RT‐PCR

mRNAs were reverse transcribed using the Super Script III kit (Cat. no. 18080‐051, Invitrogen) according to the manufacturer's recommendations. Expression of human and murine *PTPN6* gene were measured in a quantitative PCR 7500 Fast real time PCR instrument (Thermofisher©) using the TaqMan universal PCR Master Mix (Cat. no. 4304437, Invitrogen©) under the following conditions: 50°C for 2 min, 95°C for 10 min, 95°C for 10 s and 60°C for 1 min. The expression of target gene was quantified after normalization to the housekeeping gene *TBP* using the ΔΔCt method. All the predesigned real‐time PCR assays were obtained from Thermofisher as follows. Human *PTPN6* (ID: Hs00169359_m1); human *TBP* (ID: Hs00427620_m1); murine *PTPN6* (ID Mm00469153_m1) and murine *TBP* (ID: Mm01277042_m1).

### 2.5 Methylation profiling

The methylation profile of the SHP‐1 promoter was analysed after bisulfite conversion of DNA extracted from UT‐7 JAK2WT or JAK2^V617F^ by Active Motif©. PCR primers to the target region were designed with the MethPrimer software (http://www.urogene.org/cgibin/methprimer/methprimer.cgi). Primers were used to amplify the target region from bisulfite converted genomic DNA, and processed into standard, barcoded Illumina sequencing libraries and sequenced in NextSeq 500. Reads were analysed using the bismark alignment program (v 0.7.7) (http://www.bioinformatics.babraham.ac.uk/projects/bismark/). As reference sequence, human chr12 (hg19 assembly) was used. Bismark alignment reports are compiled in the file ‘4373Inserm bismark reports.xlsx’. 5.7 and 4.8 million reads were analysed for two samples.

### 2.6 Cell transduction

The SHP‐1 cDNA plasmid ‘SHP1 (PTPN6) Human Tagged ORF Clone’ for overexpression of SHP‐1 was purchased from Origene (clone no RC213896L4). Empty cDNA vector ‘pLenti‐C‐mGFP‐P2A‐Puro Lentiviral Gene Expression Vector’ for plasmid control was also purchased from Origene (Clone no PS100093). Both plasmids carry a chloramphenicol resistance gene. The lentiviral envelope plasmid VSVG (pmEOS‐2VSVG) and packaging plasmid PAX2 (pCMV‐deltaR8.2) were obtained from Addgene.

The plasmids were transformed into the Stbl3 strain (Invitrogen C7373‐03) following the manufacturer's instruction and 250 μL of pre‐warmed SOC medium were added to each vial. After 1 h shaking at 37°C, and overnight culture in petri dish at 37°C, colonies were transferred into LB medium supplemented with the corresponding antibiotic for 24 h later. Plasmid DNA was extracted and purified from the bacteria using Hispeed plasmid Maxi kit from Qiagen (Cat. no: 12663) and following manufacturer's protocol. Transfection of HEK293T cells was performed in an L3 facility in order to produce lentiviral vectors that were later transduced into the target cells. 1 × 10^6^ cells (HEL or UKE‐1) were transduced by viral culture supernatant with polybrene. Spinoculation was done at a slow rate centrifugation (900 rpm) for 1 h 30 min. The pellet was re‐suspended with the supernatant and the transduced cells were cultured in the appropriate medium devoid of any antibiotic for 48 h before collection. GFP‐positive transduced cells were then sorted using a FACS cell sorter (FACS Aria III (BD)). For experiments comparing cells transduced with the WT or mutated SHP‐1 cDNA, the viral suspensions were titrated using the in order to add the same quantity of virus. Before each experiment, the transduced cells were FACS‐sorted to have more than 90% GFP‐positive cells and hence comparable batches of cells.

### 2.7 Directed mutagenesis

The Cysteine 453 in the phosphatase catalytic site of SHP‐1 is essential and can be targeted for enzymatic activity inhibition.^17^ Directed mutagenesis was conducted on the SHP‐1 cDNA plasmid (clone no RC213896L4) to induce a Cysteine‐to‐Serine change at amino‐acid 453 and create a new SHP‐1 inactive plasmid utilizing the QuikChange Lightning Site‐Directed Mutagenesis Kit (Cat. no. 210518 from Agilent Technologies). The mutagenic primers were designed using the ‘web based QuikChange Primer Design Program’ to generate forward (ATGCCGGCGCTGCTGTGCACGATGATG) and reverse primers (CATCATCGTGCACAGCAGCGCCGGCAT). The reaction was performed with 50 ng of plasmid: 2 min at 95°C, followed by 18 cycles of 20 s at 95°C, 10 s at 60°C, and 90 s at 68°C, and a 5‐min incubation at 68°C. The resulting PCR product was then incubated with 2 μL of Dpn1 enzyme for 5 min at 37°C, in order to digest the parental DNA. The new plasmid was verified by Sanger sequencing to validate the presence of the mutation.

## 3 RESULTS

### 3.1 TGF‐β mediated signalling and proliferation inhibition is intrinsic in JAK2^V617F^ mutated cells

**FIGURE 1:**
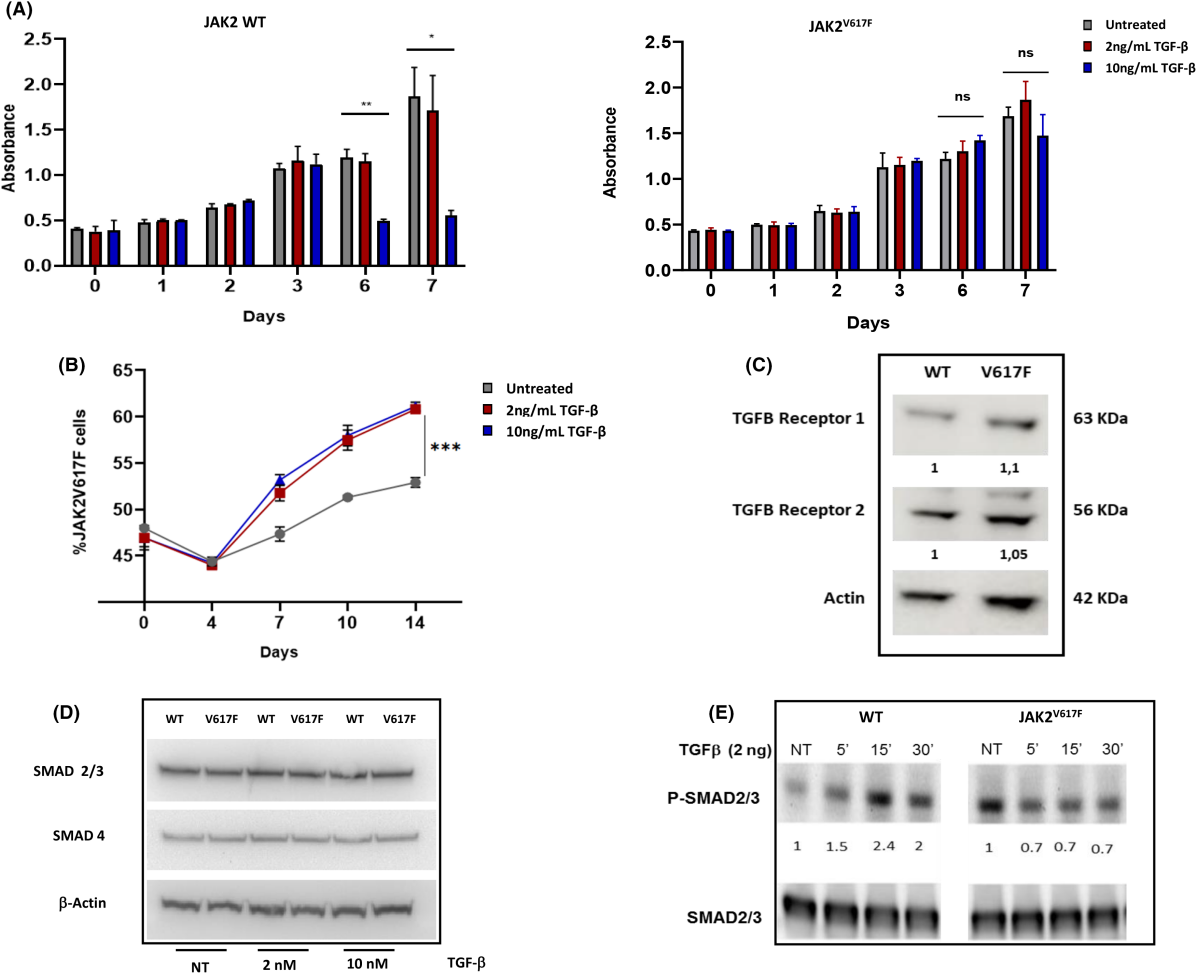
JAK2^V617F^‐mutated cells are resistant to TGF‐β. We used an isogenic model of UT‐7 cells transduced with JAK2 WT or JAK2^V617F^. (A) The proliferation of WT cells (left panel) or mutant cells (right panel) was followed during 7 days in the presence of 2 or 10 ng/mL of TGF‐β (three independent experiments). (B) JAK2^V617F^ mutant cells were transduced with an m‐cherry fluorescent reporter while the WT cells were transduced with the empty vector and a competitive experiment was performed by mixing both WT and mutant cells. The ratio of mutant to WT cells was measured by flow cytometry for 2 weeks. One representative experiment out of three. Western blot analysis of total protein extracts from UT‐7 cells expressing WT or mutated JAK2 to study the expression of: TGF‐β receptor I (C); TGF‐β receptor II (C); SMAD proteins (D) or the phosphorylated forms of SMAD2/3 activating proteins after treatment with TGF‐β (E) (one representative experiment out of three) *: *p* < 0.05, **: *p* < 0.01, ***: *p* < 0.001.

To decipher whether TGF‐β resistance of JAK2^V617F^ mutated cells is related to extrinsic or intrinsic mechanisms, we used isogenic megakaryoblastic UT‐7 cell lines differing only in the presence of a mutant or a wild‐type (WT) form of JAK2 and analysed their proliferation when high dose of active TGF‐β was added in culture. TGF‐β 10 ng/mL reduced the proliferation of WT cells by 65% and 80% after 6 and 7 days. In contrast, the growth of JAK2^V617F^ cells was unchanged (Figure 1A). We launched a competitive experiment using a mixture of 60% WT and 40% JAK2^V617F^ UT‐7 cells cultured in the presence of TGF‐β or not. We measured the proportions of mutant to WT cells by flow cytometry for 2 weeks (JAK2^V617F^ UT‐7 cells transduced with an m‐cherry fluorescent reporter while WT cells transduced with the empty vector). In line with the resistance to TGF‐β of JAK2^V617F^ mutant cells, we observed a progressive increase in the ratio of mutant to WT cells in the presence of this cytokine (Figure 1B). Notably, in the absence of TGF‐β the JAK2 mutant cells had only a slight proliferative advantage over WT cells. These results suggest that TGF‐β is important in the process of selection of the JAK2^V617F^ cells during MPN development and that resistance of JAK2^V617F^ cells to inhibition by TGF‐β is an intrinsic mechanism. Interestingly using a low dose of TGF‐β (2 ng/mL) in WT cells did not reduce the proliferation while 10 ng/mL concentration was efficient. These results may suggest that the JAK2^V617F^‐induced intrinsic defect could be due to a lower expression of TGF‐β signalling proteins when the mutation is present.

### 3.2 Expression of TGF‐β mediated signalling proteins are not impaired in JAK2^V617F^ mutated cells

TGF‐β receptor 1, TGF‐β Receptor II, Smad 2/3 and 4, the main proteins involved in the canonical transduction pathway of TGF‐β were studied in JAK2^V617F^ and JAK2 WT expressing cells. We found similar levels of expression of both TGF‐β receptor 1 and 2 but also of SMAD2/3 and 4 in the JAK2WT or JAK2^V617F^ mutated cells on steady‐state (Figure 1C,D). Notably, after the cells were treated with TGF‐β for 1 h, SMAD2/3 phosphorylation was increased in the JAK2WT cells while no phosphorylation was induced in JAK2^V617F^ cells (Figure 1E). This result argues for a JAK2^V617F^‐induced resistance to the TGF‐β signalling via the disruption of the functions of signalling regulators. Interestingly, when looking at basal level before TGF‐β stimulation, SMAD2/3 appears to be more phosphorylated in JAK^2V617F^ cells, suggesting a constitutive activation of this pathway that could not be further stimulated.

### 3.3 The expression of the SHP‐1 phosphatase is reduced in JAK2^V617F^ mutated cells

**FIGURE 2:**
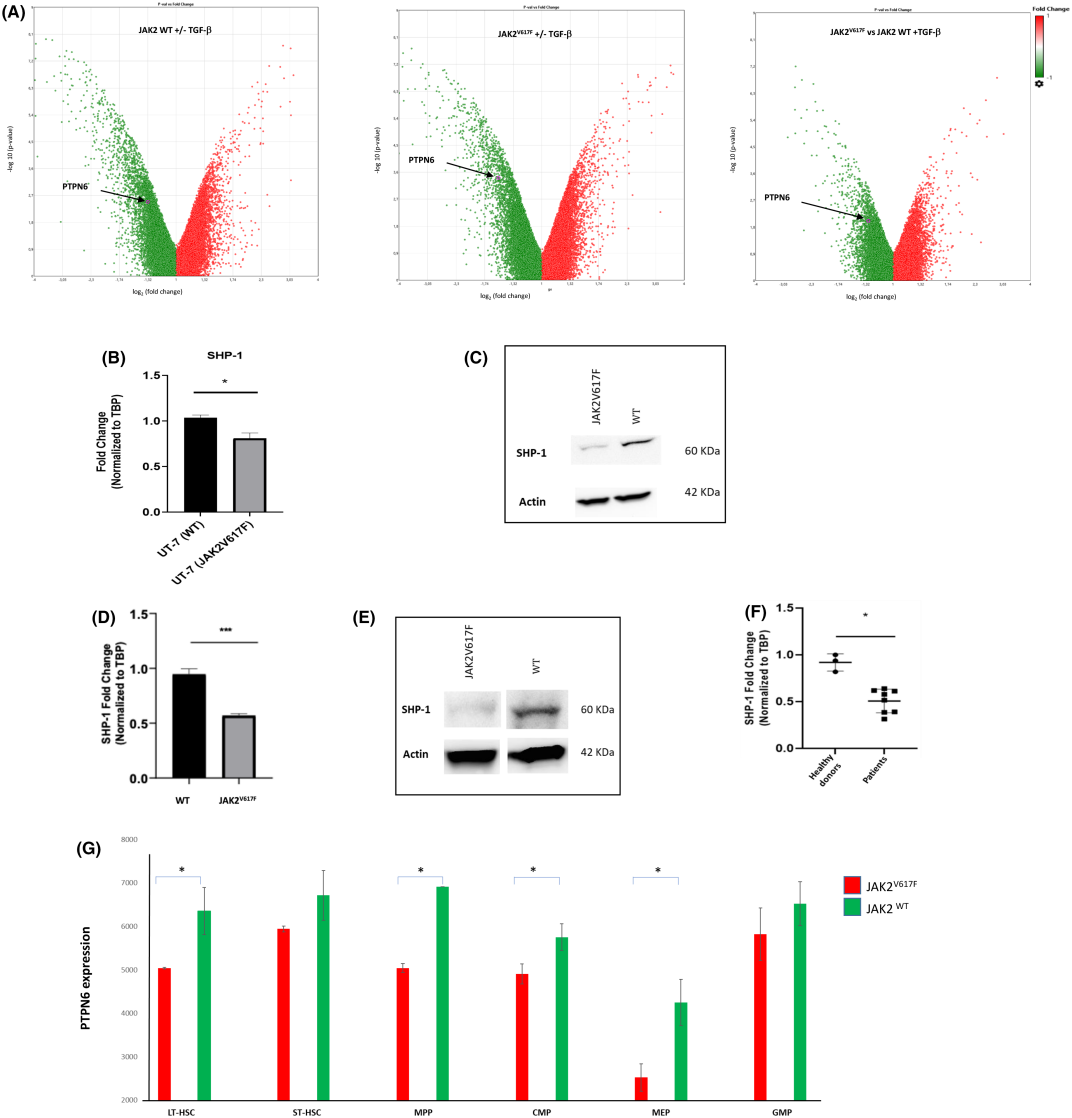
Expression of SHP‐1 is reduced in JAK2^V617F^ mutated cells. (A) Volcano plot obtained by comparing RNA‐seq data from UT‐7 cells (4 replicates) with or without treatment with TGF‐β. Left panel: JAK2 WT cells; middle panel: JAK2^V617F^ cells; right panel: Volcano plot comparing WT and JAK2^V617F^ cells under TGF‐β. (B) RT‐qPCR analysis (three independent experiments) of the expression of PTPN6/SHP‐1 in UT‐7 cells expressing WT or mutated JAK2. (C) Western blot analysis of SHP‐1 in UT‐7 cells expressing WT or mutated JAK2 (one representative experiment out of 2). (D) RT‐qPCR analysis (three independent experiments) of the expression of PTPN6/SHP‐1 in total bone marrow cells from JAK2^V617F^ knock‐in mice or syngenic WT mice. (E) Western blot analysis of SHP‐1 in total bone marrow cells from JAK2^V617F^ knock‐in mice or syngenic WT mice. (one representative experiment out of 2). (F) RT‐qPCR analysis (two independent experiments) of the expression of PTPN6/SHP‐1 in CD34 positive cells isolated from MPN patients or healthy controls, normalized to the expression of the housekeeping gene TBP. (G) PTPN6/SHP‐1 expression in RNA‐seq data obtained from purified sub‐populations of HSPCs from JAK2^V617F^ knock‐in mice or syngenic WT mice.^17^

In order to define which TGF‐β transduction pathway regulator could be deregulated in JAK2^V617F^ cells, we performed an RNA‐seq analysis in the isogenic model of the UT‐7 cell line JAK2^V617F^ or JAK2 WT after the cells were treated with TGF‐β. We focused on genes involved in the regulation of signalling pathways and identified the *PTPN6* gene, coding for the SHP‐1 phosphatase, whose expression was reduced in TGF‐β treated cells whatever the status of JAK2 (Figure 2A). The down‐regulation of SHP‐1 was even more pronounced in JAK2^V617F^ cells compared to WT cells (Figure 2A) suggesting a physiological down regulation process implicating TGF‐β and SHP‐1. To confirm the impact of the JAK2^V617F^ mutation on *PTPN6* expression, we performed RT‐qPCR and western blot on mRNA and protein extracts from JAK2WT or JAK2 mutant UT‐7 cells. The results showed that even in the absence of TGF‐β the expression of *PTPN6*/SHP‐1 was reduced at the mRNA and protein levels in the presence of the mutation (Figure 2B,C). A lower expression of SHP‐1 was also found at the mRNA and protein levels in the bone marrow cells from JAK2^V617F^ knock‐in mice compared to their wild‐type counterpart of similar ages (Figure 2D,E). The expression of the *PTPN6* gene was also significantly reduced in CD34‐positive cells isolated from the blood of JAK2^V617F^‐mutated MPN patients (1 ET, 4 PV, 3 MF) compared to healthy donors of similar ages (Figure 2F). To go further, we analysed transcriptomic data generated in a previous study from the FACS‐sorted sub‐populations of murine JAK2 mutant or WT HSPCs.^18^ The decreased expression of the *PTPN6* gene was consistently found in JAK2^V617F^‐mutated long‐term haematopoietic stem cells (LT‐HSC) and the more committed progenitors such as MPP, CMP and MEP (Figure 2G).

### 3.4 Reduced expression of SHP‐1 in JAK2^V617F^ mutated cells is related to JAK/STAT over‐signalling

**FIGURE 3:**
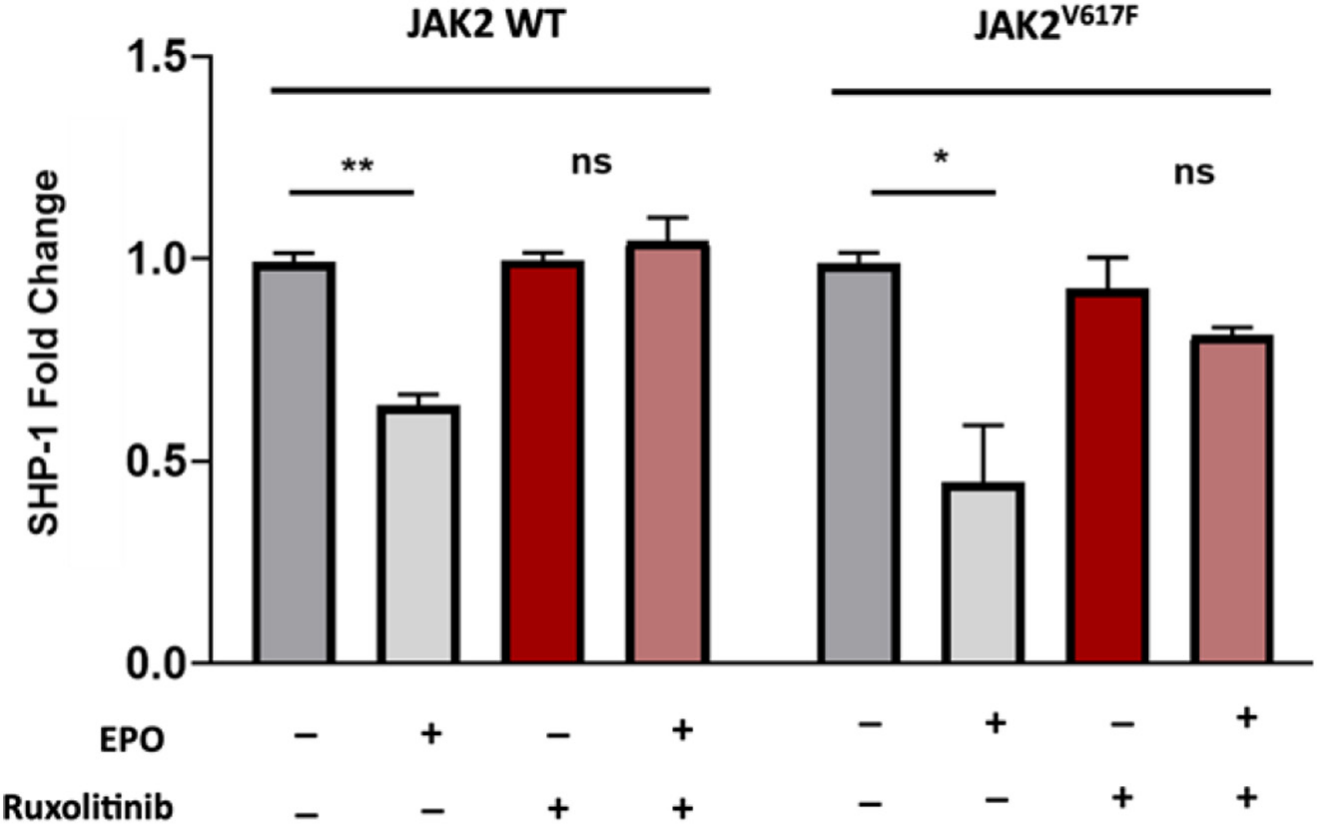
The expression of SHP‐1 is regulated by the EPO‐induced JAK2 signalling. RT‐qPCR analysis (three independent experiments) of SHP‐1 expression in UT‐7 cells expressing WT or mutated JAK2 treated with EPO (1 IU/mL) and/or the JAK2 inhibitor ruxolitinib (250 nM). *: *p*<0.05, **: *p*<0.01.

To explore whether JAK2 signalling participates in the regulation of SHP‐1 expression, we compared its expression in UT‐7 cells treated or not with EPO. The results showed that SHP‐1 expression is down regulated after EPO treatment of UT‐7 cells whatever the JAK2 wild‐type or mutant status, suggesting that *PTPN6*/SHP‐1 expression is JAK2 dependent. To confirm this JAK2 dependency of SHP‐1 expression, a pre‐treatment with the JAK2 inhibitor Ruxolitinib was performed and then EPO was added. The JAK2 inhibitor drastically abolished the effect of EPO suggesting that the EPO/JAK2 signalling is involved in SHP‐1 expression (Figure 3). Thus, the EPO/JAK2 signalling is involved in the down regulation of *PTPN6*/SHP‐1 expression, which correlates with a basal decreased expression of SHP‐1 in JAK2^V617F^‐mutated cells where the JAK/STAT signalling is constitutively activated. In contrast, specific inhibitors of the PI3Kinase and MEK/ERK MAPKinase pathways did not reproduce the effect of ruxolitinib (Figure S1), suggesting that these pathways are not involved in the regulation of the SHP‐1 expression downstream of EPO.

**FIGURE 4:**
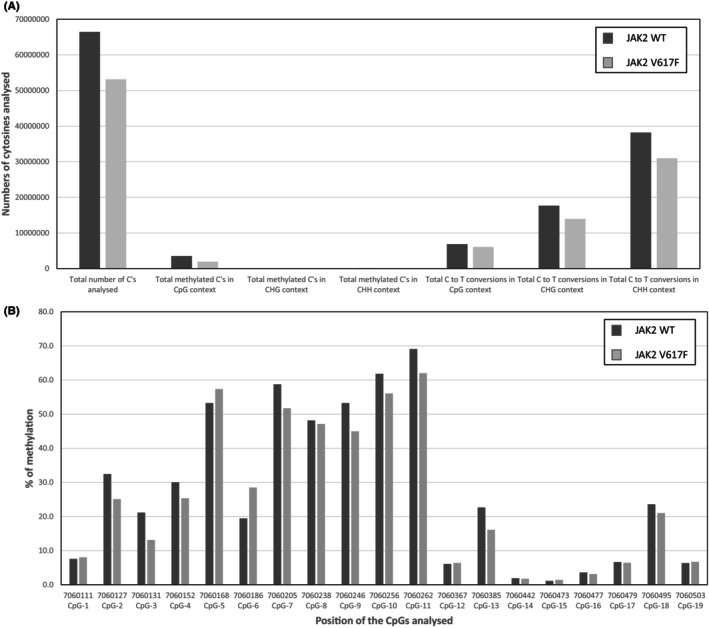
Methylation profiles of the SHP‐1 promoter in JAK2 WT or JAK2^V617F^ UT‐7 cells. (A). Global cytosine methylation results. (B) Percentages of methylated cytosines at CpGs located in the SHP‐1 promoter in UT‐7 WT of JAK2^V617F^ cell lines using three replicates. The levels of methylation do not significantly change between the two samples, showing that both samples show the same pattern of hyper‐ and hypo‐methylated sites.

A hypermethylation of the *PTPN6* promoter has been reported in leukaemic cells with reduced SHP‐1 expression including Chronic myeloid leukaemia.^19^, ^20^ However, we failed to identify any abnormal methylation of the *PTPN6* promoter in UT‐7 cells transduced with JAK2^V617F^ compared to those transduced with JAK2WT using directed analysis of CpGs after bisulfite treatment (Active Motif©) (Figure 4 and Figure S2). Furthermore, when JAK2^V617F^ or JAK2WT UT‐7 cells were treated with the demethylating agent 5‐azacytidine, we observed a similar increase in SHP‐1 expression in both cells, suggesting a similar level of *PTPN6* promoter methylation whatever JAK2 is mutated or not (Figure S3). Thus, this reinforces the hypothesis of a direct regulation of SHP‐1 expression via JAK/STAT oversignaling.

### 3.5 SHP‐1 expression correlates with the resistance of JAK2^V617F^ mutated cells to TGF‐β

**FIGURE 5:**
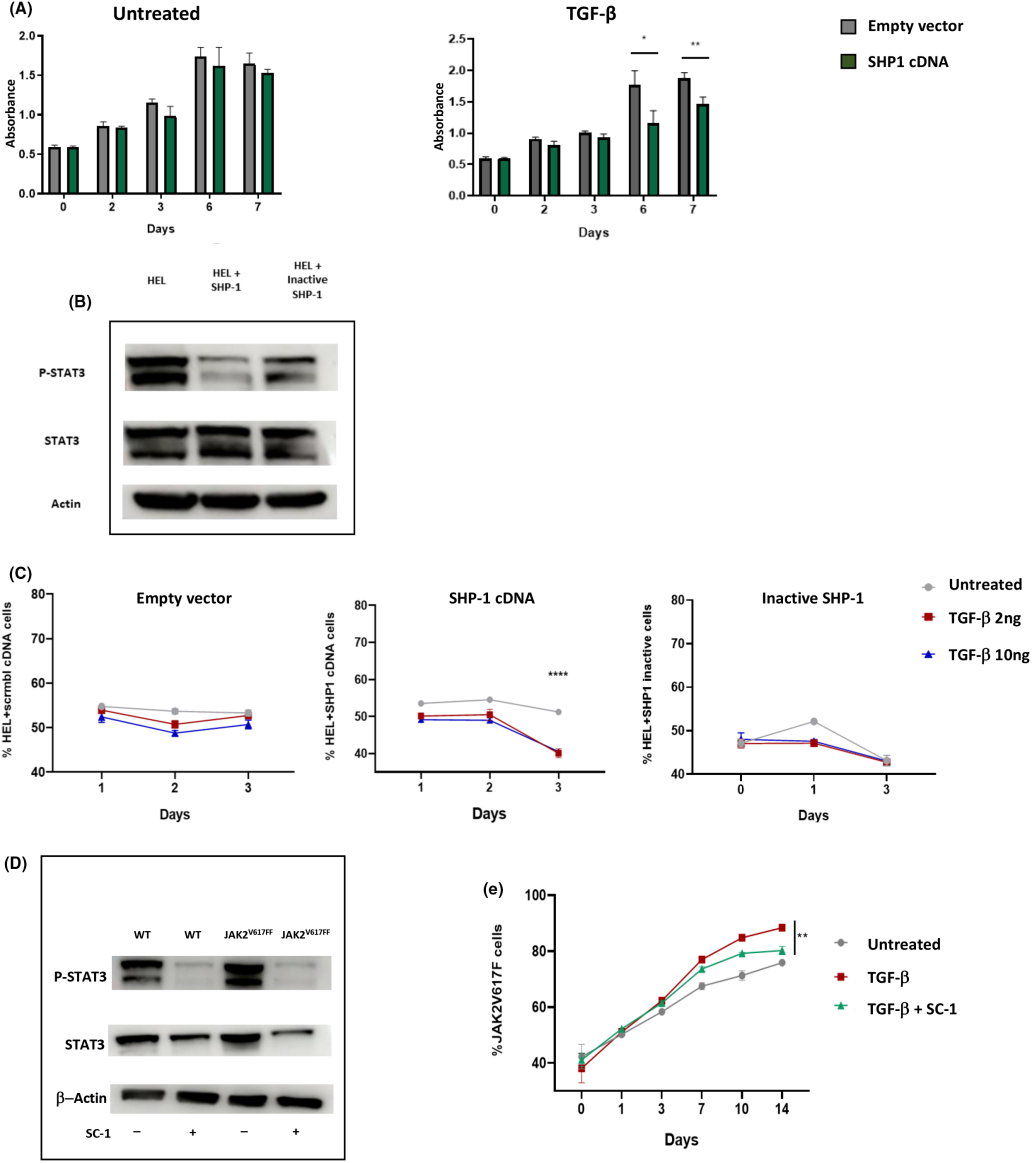
SHP‐1 expression correlates with the sensitivity to TGF‐β. (A) The JAK2^V617F^ mutated HEL cells were transduced with SHP‐1 cDNA or an empty vector and the proliferation was followed for 7 days under treatment with TGF‐β (right panel) or without treatment (left panel) (three independent experiments). (B) An enzymatically inactive form of SHP‐1 was generated by directed mutagenesis. Western blot analysis shows that STAT3 phosphorylation in HEL cells is more reduced when transduced with a WT SHP‐1 compared to the phosphatase‐inactive SHP‐1 (Cys453Ser) (one representative experiment out of 2). (C) competitive experiments were launched with HEL cells transduced with either normal SHP‐1 (middle panel), inactive SHP‐1 due to directed mutagenesis on Cysteine 453 in the active site (right panel) or empty vector (left panel). One representative experiment out of three. (D) Western blot analysis of STAT3 and phospho‐STAT3 in HEL cells treated or not with the SHP‐1 agonist SC‐1 (one representative experiment out of 2). (E) Competitive experiment between m‐cherry transduced UT‐7 JAK2^V617F^ cells and WT cells treated or not with TGF‐β 10 ng/mL and the SHP‐1 agonist SC‐1 1 μM. One representative experiment out of three. *: *p* < 0.05, **: *p* < 0.01.

To explore the relationship between SHP‐1 and the resistance of JAK2^V617F^ mutated cells to TGF‐β we lentivirally transduced the cDNA coding for SHP‐1 into the erythroleukemia cell line HEL, which carries several copies of the JAK2^V617F^ allele. HEL cells were transduced with a GFP‐tagged vector containing the human SHP‐1 cDNA or an empty vector. After FACS sorting to reach at least 90% GFP‐positive cells, we cultured the cells with or without TGF‐β (10 ng/mL) for 7 days. By measuring the proliferation rate we showed that increasing the expression of SHP‐1 in HEL cells significantly restored the sensitivity to the antiproliferative activity of TGF‐β. Indeed, in the presence of TGF‐β we observed a significant reduction in the proliferation of SHP‐1‐transduced HEL cells compared to the control cells (Figure 5A). In the absence of TGF‐β the growth was similar in both cells suggesting that SHP‐1 does not exert antiproliferative activity on its own in this context. The same results were obtained using UKE‐1, another JAK2^V617F^‐mutated cell line (Figure S4). Altogether, these results show that the resistance of JAK2^V617F^ mutated cells to the antiproliferative activity of TGF‐β is dependent on the level of expression of SHP‐1.

### 3.6 SHP‐1 phosphatase activity is responsible for the resistance to TGF‐β of JAK2^V617F^ mutated cells

To question the mechanism through which SHP‐1 is involved in the resistance of JAK2^V617F^ mutated cells to TGF‐β, we performed a directed mutagenesis in the SHP‐1 cDNA to change the catalytically active cysteine 453 for a serine in the catalytic site of the enzyme.^17^ We confirmed that the SHP‐1 mutant is phosphatase‐defective as it was significantly less effective than wild‐type SHP‐1 to reduce the phosphorylation of STAT3 (Figure 5B), a SHP‐1 target in haematopoietic cells.^21^ Then we performed a competitive assay under TGF‐β treatment between cells transduced with a phosphatase active or inactive SHP‐1. Of note, we verified by RT‐qPCR that cells transduced with the WT or the inactive form of SHP‐1 express similar levels of SHP‐1 (Figure S5). The results showed a loss in the restoration of sensitivity to the antiproliferative activity of TGF‐β when SHP‐1 is phosphatase‐defective (Figure 5C). This strongly suggests that the main consequence of the down‐regulation of SHP‐1 expression leading to the selection of JAK2^V617F^‐mutated cells in the presence of TGF‐β is the global intracellular decrease in its enzymatic activity. To confirm our findings, we performed a pre‐clinical approach aiming at counteracting the selection of JAK2^V617F^‐mutated cells through the increase in SHP‐1 enzymatic activity. Small molecule SHP‐1 agonists have been developed such as the sorafenib derivative SC‐1.^22^ We confirmed the potential of SC‐1 as a SHP‐1 agonist in HEL cells, as it is able to increase the dephosphorylation of STAT3 (Figure 5D). Then we tested whether SC‐1 may counteract the TGF‐β dependent selection of JAK2^V617F^‐mutated cells. For that purpose, we performed a competition assay using UT‐7 cells as described above, in which a mixture of 60% JAK2WT and 40% JAK2^V617F^ cells was incubated with TGF‐β and SC‐1. We observed that SC‐1 significantly antagonized the selection of JAK2 mutated cells (Figure 5E) while we confirmed the role of TGF‐β in the selection of these cells. Altogether, our results demonstrate that the enzymatic activity is necessary for the SHP‐1‐dependent antiproliferative activity of TGF‐β in the context of JAK2^V617F^‐mutated cells. Accordingly, we validated the potential of SHP‐1 agonists to counteract the selection of oncogenic cells in the context of MPN.

## 4 DISCUSSION

MPNs are haematopoietic malignancies initiated by the acquisition at the HSPC level of mutations activating the JAK/STAT pathway. The most frequent mutation is JAK2^V617F^ and the molecular follow‐up of this mutation in MPN patients usually shows a regular increase of the allelic frequency which correlates with the clinical evolution. The progressive clonal selection of JAK2^V617F^ cells is also confirmed in mice models. When both wild type and JAK2^V617F^ cells are co‐transplanted, the mutant chimerism in recipient mice is increasing during time post‐transplantation. Indeed, as exemplified by single cell transplantation studies in mice,^3^ JAK2^V617F^‐mutated cells progressively outcompete JAK2 wild type cells to progressively invade bone marrow and peripheral blood. The mechanisms of this clonal selection are incompletely identified, and probably are due to cell intrinsic and extrinsic factors. One of the consequences of the JAK2^V617F^ mutation in haematopoietic progenitors is the increased production of TGF‐β in the bone marrow microenvironment, which is associated with the secondary development of myelofibrosis.^10^ However, among the pleiotropic effects of TGF‐β is its role in the negative regulation of HSPCs proliferation, as it is considered essential for maintaining their quiescence. Although resistance to TGF‐β in MPN cells has long been described^23^, ^24^ no clear mechanism has been proposed with the exception of reduced expression of SMAD4 in ET patient cells^25^ or reduction of TGFbRII mRNA in CD34‐positive cells from MF patients.^24^ Specifically, the mechanisms by which JAK2^V617F^‐mutated cells escape the antiproliferative control of TGF‐β are largely unexplained. In this study we identified the down‐regulation of SHP‐1 expression in JAK2^V617F^‐mutated cells in both cell lines, murine cells and primary MPN patients' cells, in line with the report in 1999 of a decreased SHP‐1 expression in cells from polycythemia vera patients.^26^ Interestingly in mice, SHP‐1 expression was similarly reduced in all the subtypes of HSPCs, from LT‐HSCs, CMP to MEP, suggesting it is a general consequence of this mutation in haematopoietic cells, due to a common regulatory mechanism in every cell types. We identified that the reduced expression is mainly dependent on JAK2 signalling pathway. However, how hyperactivated JAK2 signalling leads to the down‐regulation of SHP‐1 expression is not established. Indeed, unlike the hypermethylation of the *PTPN6* promoter reported in CML cells,^20^ we did not identify increased CpGs methylation in this promoter region in the JAK2^V617F^‐mutated cells. Demethylating agents are therefore unlikely to restore TGF‐β sensitivity in mutant cells without affecting normal cells. Other mechanisms of epigenetic regulation, such as changes in histone acetylation or methylation, may be responsible and should be the subject of specific studies.

SHP‐1 is involved in the regulation of several intracellular signalling pathways, such as immune regulation as initially demonstrated in SHP‐1 deficient motheaten mice.^27^ Interestingly, motheaten mice and JAK2^V617F^ knock‐in mice share common characteristics such as small size, hairlessness and chronic inflammation with increased IL‐6 and TNF‐a expression, suggesting shared dysregulations in both models.

Using overexpression of SHP‐1 and competitive experiments we obtained conclusive results supporting the role of the down‐regulation of this phosphatase in the resistance of JAK2^V617F^‐mutated cells to the antiproliferative activity of TGF‐β. This is consistent with recent studies describing the role of SHP‐1 in the regulation of intracellular pathways and the control of HSPCs quiescence by TGF‐β.^16^ In this study it was shown that SHP‐1 increases TGF‐β signalling by interacting with the ITIM domain of the TGF‐β receptor but the exact mechanism is still unexplained.^16^ Because SHP‐2, which has a high level of sequence and structure similarity with SHP‐1, is known to regulate signalling pathways through either protein interaction and/or through its phosphatase activity, we developed a phosphatase‐defective model of SHP‐1. This allowed us to show that the loss of sensitivity to TGF‐β observed in JAK2^V617F^‐mutated cells is due to the loss in SHP‐1 phosphatase enzymatic activity. The hyperactivation of intracellular signalling pathways is a hallmark of cancer cells which can be due to a variety of causes: mutations or chromosomal rearrangements targeting tyrosine kinases (TK) such as FLT3, KIT or BCR::ABL1 alterations in hematologic malignancies; indirect mechanisms such as HER2 TK receptor overexpression in breast cancers; mutations in PI3K/AKT or RAS signalling pathways in many cancer subtypes. Because the most frequent consequence is the dysregulation of kinase activity, a large amount of research has been undertaken to develop kinase inhibitors in the field of cancer, with impressive success once transferred to the clinic.^28^ However, complementary approaches by activating phosphatases to negatively regulate signalling pathways have not been as largely studied and only few phosphatase activators are available. SHP‐1 agonists were developed such as SC‐1, a derivative of the pan kinase inhibitor sorafenib which is able to inhibit TGF‐β signalling and to reduce liver and kidney fibrosis development.^29^, ^30^ We confirmed that SC‐1 increases SHP‐1 activity in haematopoietic cells as it reduces STAT3 phosphorylation. Of note, based on the oncogenic role of STAT3, preclinical studies showed the therapeutic potential of SHP‐1 agonists in models of hepatocarcinoma.^31^ Using SC‐1 restored the sensitivity to TGF‐β in JAK2^V617F^‐mutated cells and hampered the selection of these cells in competitive experiments, when exposed to TGF‐β. This confirmed that the reduction of SHP‐1 activity is one of the mechanisms explaining the clonal selection of JAK2^V617F^‐mutated cells in MPN.

It is well accepted that MPN development and evolution are largely due to clonal selection.^32^ Also, JAK2^V617F^ mutation is one of the most frequent mutations identified in people with clonal haematopoiesis of indeterminate potential (CHIP)^33^ and it is well described that MPN patients may harbour the mutation decades before the onset of the disease.^4^, ^34^, ^35^ Therefore, SHP‐1 agonists may be a new therapeutic approach in these diseases to antagonize the clonal advantage of JAK2^V617F^ mutated cells and avoid the development of secondary MPN in people with CHIP.

## AUTHOR CONTRIBUTIONS

**Céline Aoun:** Formal analysis (equal); investigation (equal); validation (equal); visualization (equal); writing – original draft (equal). **Nabih Maslah:** Investigation (equal); resources (equal); visualization (equal); writing – review and editing (equal). **Saravanan Ganesan:** Investigation (equal); methodology (equal); resources (equal); writing – review and editing (equal). **Norman Salomao:** Investigation (equal); writing – review and editing (equal). **Romane Gendron:** Investigation (equal); writing – review and editing (equal). **Sarah Awan Toor:** Investigation (equal); writing – review and editing (equal). **Gil Letort:** Investigation (equal); writing – review and editing (equal). **Panhong Gou:** Investigation (equal); writing – review and editing (equal). **Mélina Bonnamy:** Investigation (equal); writing – review and editing (equal). **Véronique Parietti:** Methodology (equal); resources (equal); supervision (equal); writing – review and editing (equal). **Jean‐Jacques Kiladjian:** Conceptualization (equal); funding acquisition (lead); methodology (equal); writing – review and editing (equal). **Stephane Giraudier:** Conceptualization (equal); formal analysis (equal); resources (equal); supervision (equal); visualization (equal); writing – original draft (equal). **Bruno Cassinat:** Conceptualization (lead); formal analysis (equal); funding acquisition (equal); methodology (equal); resources (equal); supervision (lead); writing – original draft (lead).

## FUNDING INFORMATION

The study was supported by grants from ‘INCa Prev‐Bio 2021’, ‘Fondation ARC pour la recherche sur le cancer’ and ‘Fondation leucémie espoir’ fundings. C. Aoun was supported during her PhD thesis by a grant from ED565 ‘Hematologie, Oncologie et Biotherapies’ and also from ‘Fondation ARC pour la recherche sur le cancer’.

## CONFLICT OF INTEREST STATEMENT

The authors have no conflicts of interest to disclose.

## Supporting information


Figure S1.

Figure S2.

Figure S3.

Figure S4.

Figure S5.

Table S1.

## Data Availability

The data that support the findings of this study are available from the corresponding author upon reasonable request.

## References

[1] Vainchenker W , Kralovics R . Genetic basis and molecular pathophysiology of classical myeloproliferative neoplasms. Blood. 2017;129(6):667‐679. 28028029 10.1182/blood-2016-10-695940

[2] Jamieson CH , Gotlib J , Durocher JA , et al. The JAK2 V617F mutation occurs in hematopoietic stem cells in polycythemia vera and predisposes toward erythroid differentiation. Proc Natl Acad Sci USA. 2006;103(16):6224‐6229. 16603627 10.1073/pnas.0601462103 PMC1434515

[3] Lundberg P , Takizawa H , Kubovcakova L , et al. Myeloproliferative neoplasms can be initiated from a single hematopoietic stem cell expressing JAK2‐V617F. J Exp Med. 2014;211(11):2213‐2230. 25288396 10.1084/jem.20131371 PMC4203945

[4] Williams N , Lee J , Mitchell E , et al. Life histories of myeloproliferative neoplasms inferred from phylogenies. Nature. 2022;602(7895):162‐168. 35058638 10.1038/s41586-021-04312-6

[5] Hasan S , Lacout C , Marty C , et al. JAK2V617F expression in mice amplifies early hematopoietic cells and gives them a competitive advantage that is hampered by IFNα. Blood. 2013;122(8):1464‐1477. 23863895 10.1182/blood-2013-04-498956

[6] Nienhold R , Ashcroft P , Zmajkovic J , et al. MPN patients with low mutant JAK2 allele burden show late expansion restricted to erythroid and megakaryocytic lineages. Blood. 2020;136(22):2591‐2595. 32698197 10.1182/blood.2019002943

[7] Koschmieder S , Chatain N . Role of inflammation in the biology of myeloproliferative neoplasms. Blood Rev. 2020;42:100711. 32505517 10.1016/j.blre.2020.100711

[8] Mendez Luque LF , Blackmon AL , Ramanathan G , Fleischman AG . Key role of inflammation in myeloproliferative neoplasms: instigator of disease initiation, progression and symptoms. Curr Hematol Malig Rep. 2019;14(3):145‐153. 31119475 10.1007/s11899-019-00508-w PMC7746200

[9] Heyde A , Rohde D , McAlpine CS , et al. Increased stem cell proliferation in atherosclerosis accelerates clonal hematopoiesis. Cell. 2021;184(5):1348‐1361. 33636128 10.1016/j.cell.2021.01.049 PMC8109274

[10] Chagraoui H , Komura E , Tulliez M , Giraudier S , Vainchenker W , Wendling F . Prominent role of TGF‐beta 1 in thrombopoietin‐induced myelofibrosis in mice. Blood. 2002;100(10):3495‐3503. 12393681 10.1182/blood-2002-04-1133

[11] Vannucchi AM , Bianchi L , Paoletti F , et al. A pathobiologic pathway linking thrombopoietin, GATA‐1, and TGF‐beta1 in the development of myelofibrosis. Blood. 2005;105(9):3493‐3501. 15665119 10.1182/blood-2004-04-1320

[12] Sitnicka E , Ruscetti FW , Priestley GV , Wolf NS , Bartelmez SH . Transforming growth factor beta 1 directly and reversibly inhibits the initial cell divisions of long‐term repopulating hematopoietic stem cells. Blood. 1996;88(1):82‐88. 8704205

[13] Batard P , Monier MN , Fortunel N , et al. TGF‐(beta)1 maintains hematopoietic immaturity by a reversible negative control of cell cycle and induces CD34 antigen up‐modulation. J Cell Sci. 2000;113(3):383‐390. 10639326 10.1242/jcs.113.3.383

[14] Yamazaki S , Iwama A , Takayanagi S , Eto K , Ema H , Nakauchi H . TGF‐beta as a candidate bone marrow niche signal to induce hematopoietic stem cell hibernation. Blood. 2009;113(6):1250‐1256. 18945958 10.1182/blood-2008-04-146480

[15] Ciurea SO , Merchant D , Mahmud N , et al. Pivotal contributions of megakaryocytes to the biology of idiopathic myelofibrosis. Blood. 2007;110(3):986‐993. 17473062 10.1182/blood-2006-12-064626 PMC1924766

[16] Jiang L , Han X , Wang J , et al. SHP‐1 regulates hematopoietic stem cell quiescence by coordinating TGF‐β signaling. J Exp Med. 2018;215(5):1337‐1347. 29669741 10.1084/jem.20171477 PMC5940262

[17] Kosugi A , Sakakura J , Yasuda K , Ogata M , Hamaoka T . Involvement of SHP‐1 tyrosine phosphatase in TCR‐mediated signaling pathways in lipid rafts. Immunity. 2001;14(6):669‐680. 11420038 10.1016/s1074-7613(01)00146-7

[18] Gou P , Liu D , Ganesan S , et al. Genomic and functional impact of Trp53 inactivation in JAK2V617F myeloproliferative neoplasms. Blood Cancer J. 2024;14(1):1. 38177095 10.1038/s41408-023-00969-6 PMC10766605

[19] Oka T , Yoshino T , Hayashi K , et al. Reduction of hematopoietic cell‐specific tyrosine phosphatase SHP‐1 gene expression in natural killer cell lymphoma and various types of lymphomas/leukemias: combination analysis with cDNA expression array and tissue microarray. Am J Pathol. 2001;159(4):1495‐1505. 11583976 10.1016/S0002-9440(10)62535-7 PMC1850490

[20] Li Y , Liu X , Guo X , Liu X , Luo J . DNA methyltransferase 1 mediated aberrant methylation and silencing of SHP‐1 gene in chronic myelogenous leukemia cells. Leuk Res. 2017;58:9‐13. 28376405 10.1016/j.leukres.2017.03.014

[21] Dong F , Qiu Y , Yi T , Touw IP , Larner AC . The carboxyl terminus of the granulocyte colony‐stimulating factor receptor, truncated in patients with severe congenital neutropenia/acute myeloid leukemia, is required for SH2‐containing phosphatase‐1 suppression of Stat activation. J Immunol. 2001;167(11):6447‐6452. 11714811 10.4049/jimmunol.167.11.6447

[22] Su TH , Shiau CW , Jao P , et al. Sorafenib and its derivative SC‐1 exhibit antifibrotic effects through signal transducer and activator of transcription 3 inhibition. Proc Natl Acad Sci USA. 2015;112(23):7243‐7248. 26039995 10.1073/pnas.1507499112 PMC4466718

[23] Zauli G , Visani G , Catani L , Vianelli N , Gugliotta L , Capitani S . Reduced responsiveness of bone marrow megakaryocyte progenitors to platelet‐derived transforming growth factor beta 1, produced in normal amount, in patients with essential thrombocythaemia. Br J Haematol. 1993;83(1):14‐20. 8435322 10.1111/j.1365-2141.1993.tb04624.x

[24] Le Bousse‐Kerdilès MC , Chevillard S , Charpentier A , et al. Differential expression of transforming growth factor‐beta, basic fibroblast growth factor, and their receptors in CD34^+^ hematopoietic progenitor cells from patients with myelofibrosis and myeloid metaplasia. Blood. 1996;88(12):4534‐4546. 8977245

[25] Kuroda H , Matsunaga T , Terui T , et al. Decrease of Smad4 gene expression in patients with essential thrombocythaemia may cause an escape from suppression of megakaryopoiesis by transforming growth factor‐beta1. Br J Haematol. 2004;124(2):211‐220. 14687032 10.1046/j.1365-2141.2003.04755.x

[26] Wickrema A , Chen F , Namin F , et al. Defective expression of the SHP‐1 phosphatase in polycythemia vera. Exp Hematol. 1999;27(7):1124‐1132. 10390187 10.1016/s0301-472x(99)00043-0

[27] Tsui HW , Siminovitch KA , de Souza L , Tsui FW . Motheaten and viable motheaten mice have mutations in the haematopoietic cell phosphatase gene. Nat Genet. 1993;4(2):124‐129. 8348149 10.1038/ng0693-124

[28] Gharwan H , Groninger H . Kinase inhibitors and monoclonal antibodies in oncology: clinical implications. Nat Rev Clin Oncol. 2016;13(4):209‐227. 26718105 10.1038/nrclinonc.2015.213

[29] Chen YL , Lv J , Ye XL , et al. Sorafenib inhibits transforming growth factor β1‐mediated epithelial‐mesenchymal transition and apoptosis in mouse hepatocytes. Hepatology. 2011;53(5):1708‐1718. 21360571 10.1002/hep.24254

[30] Jia L , Ma X , Gui B , et al. Sorafenib ameliorates renal fibrosis through inhibition of TGF‐β‐induced epithelial‐mesenchymal transition. PLoS One. 2015;10(2):e0117757. 25679376 10.1371/journal.pone.0117757 PMC4332653

[31] Fan LC , Shiau CW , Tai WT , et al. SHP‐1 is a negative regulator of epithelial‐mesenchymal transition in hepatocellular carcinoma. Oncogene. 2015;34(41):5252‐5263. 25619838 10.1038/onc.2014.445

[32] Maslah N , Benajiba L , Giraudier S , Kiladjian JJ , Cassinat B . Clonal architecture evolution in myeloproliferative neoplasms: from a driver mutation to a complex heterogeneous mutational and phenotypic landscape. Leukemia. 2023;37(5):957‐963. 37002477 10.1038/s41375-023-01886-0 PMC10169637

[33] Jaiswal S , Fontanillas P , Flannick J , et al. Age‐related clonal hematopoiesis associated with adverse outcomes. N Engl J Med. 2014;371(26):2488‐2498. 25426837 10.1056/NEJMoa1408617 PMC4306669

[34] McKerrell T , Park N , Chi J , et al. JAK2 V617F hematopoietic clones are present several years prior to MPN diagnosis and follow different expansion kinetics. Blood Adv. 2017;1(14):968‐971. 29296738 10.1182/bloodadvances.2017007047 PMC5737599

[35] Nielsen C , Bojesen SE , Nordestgaard BG , Kofoed KF , Birgens HS . JAK2V617F somatic mutation in the general population: myeloproliferative neoplasm development and progression rate. Haematologica. 2014;99(9):1448‐1455. 24907356 10.3324/haematol.2014.107631 PMC4562533

