# Supplementary material for: JAK2V617F‐dependent down regulation of SHP‐1 expression participates in the selection of myeloproliferative neoplasm cells in the presence of TGF‐β
Source: J Cell Mol Med. 2024 Oct 21;28(20):e70138. doi: 10.1111/jcmm.70138 (PMC11492149; doi:10.1111/jcmm.70138)
Supplement: Supplementary file 1 — Figure S1. [file JCMM-28-e70138-s002.pdf]

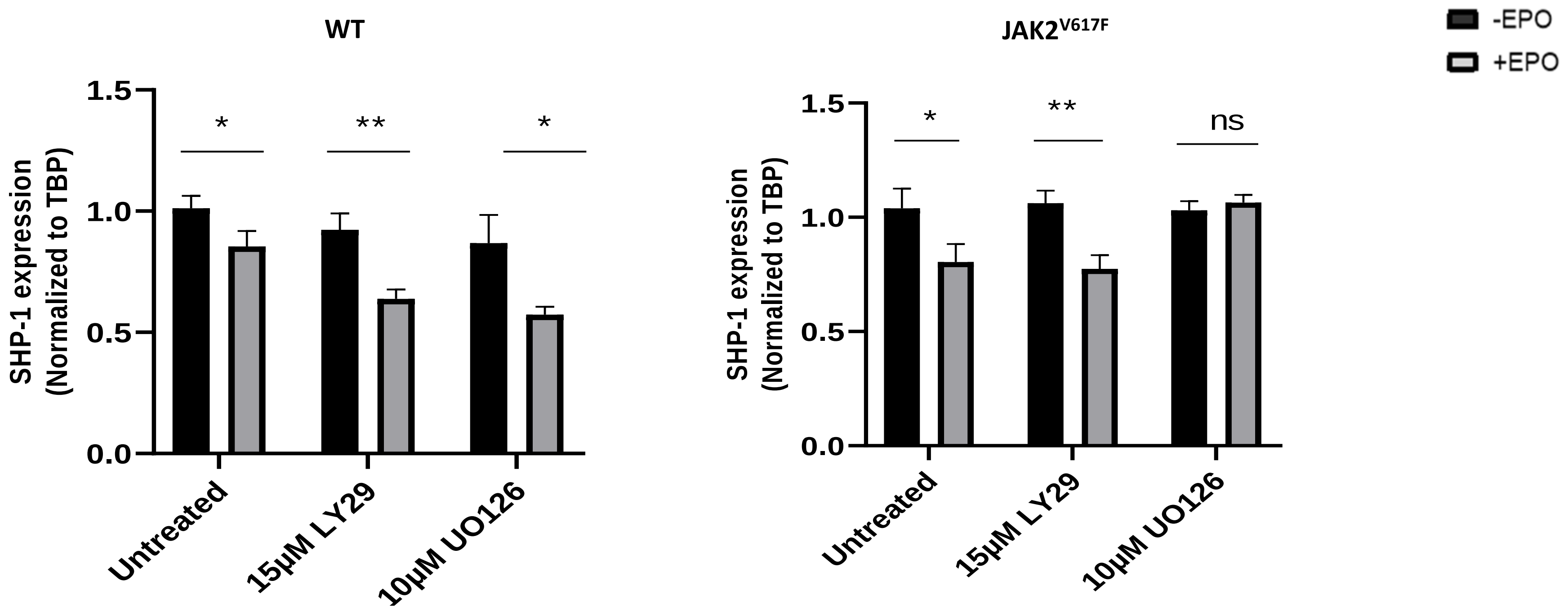

Supplementary Figure S1 : RT-qPCR analysis of SHP-1 expression in UT-7 cells expressing WT or mutated JAK2 treated with EPO and the MEK/ERK MAPKinase inhibitor UO126 or the PI3Kinase inhibitor LY294002. *: $p<0.05$, **: $p<0.01$, NS: non significant.
