# Supplementary material for: JAK2V617F‐dependent down regulation of SHP‐1 expression participates in the selection of myeloproliferative neoplasm cells in the presence of TGF‐β
Source: J Cell Mol Med. 2024 Oct 21;28(20):e70138. doi: 10.1111/jcmm.70138 (PMC11492149; doi:10.1111/jcmm.70138)
Supplement: Supplementary file 2 — Figure S2. [file JCMM-28-e70138-s006.pdf]

Supplementary Figure S2: Percentages of methylated cytosines among the promoter in both cell lines: Low methylation (0%) in blue, high methylation (>80%) in red, and intermediate values around 20% in white. As a negative control, it is expected that the non-CpG residues are not methylated. The level of methylation does not significantly change between the 2 samples showing that both samples show the same pattern of hyper- and hypo-methylated sites.

Chromosome	Position	Context	JAK2 WT	JAK2 V617F
chr12	7060062	CHH	0.1	0.2
chr12	7060063	CHG	0.0	0.1
chr12	7060069	CHH	0.2	0.4
chr12	7060076	CHG	0.2	0.4
chr12	7060089	CHH	0.0	0.1
chr12	7060090	CHH	0.3	0.2
chr12	7060094	CHG	0.1	0.3
chr12	7060098	CHH	0.0	0.1
chr12	7060103	CHH	0.4	0.4
chr12	7060111	CpG-1	7.6	8.0
chr12	7060113	CHH	0.1	0.2
chr12	7060114	CHH	0.8	0.7
chr12	7060122	CHH	0.3	0.4
chr12	7060124	CHG	0.2	0.7
chr12	7060127	CpG-2	32.4	25.1
chr12	7060129	CHH	0.2	1.2
chr12	7060130	CHG	0.1	0.2
chr12	7060131	CpG-3	21.1	13.1
chr12	7060134	CHH	0.2	0.1
chr12	7060137	CHH	0.2	0.2
chr12	7060138	CHG	0.2	0.3
chr12	7060141	CHG	0.1	0.3
chr12	7060146	CHH	0.2	0.4
chr12	7060148	CHH	0.3	0.3
chr12	7060150	CHH	0.1	0.4
chr12	7060152	CpG-4	30.0	25.3
chr12	7060158	CHG	0.1	0.5
chr12	7060165	CHH	0.6	0.6
chr12	7060167	CHG	0.3	0.6
chr12	7060168	CpG-5	53.2	57.3
chr12	7060170	CHH	0.4	0.4
chr12	7060171	CHH	0.2	0.4
chr12	7060174	CHH	0.4	0.1
chr12	7060186	CpG-6	19.4	28.5
chr12	7060192	CHH	0.2	0.2
chr12	7060193	CHG	0.2	0.9
chr12	7060202	CHG	0.2	0.8
chr12	7060205	CpG-7	58.7	51.7
chr12	7060213	CHG	0.7	0.5
chr12	7060216	CHG	0.3	0.5
chr12	7060237	CHG	0.5	0.7
chr12	7060238	CpG-8	48.2	47.1
chr12	7060245	CHG	0.2	0.2
chr12	7060246	CpG-9	53.2	45.0
chr12	7060249	CHG	0.2	0.6
chr12	7060256	CpG-10	61.8	56.1
chr12	7060258	CHH	0.5	0.5
chr12	7060262	CpG-11	69.1	62.0
chr12	7060275	CHG	0.2	0.2
chr12	7060280	CHH	0.3	0.3
chr12	7060281	CHG	0.3	0.2
chr12	7060291	CHH	0.1	0.4
chr12	7060296	CHH	0.1	0.3
chr12	7060298	CHH	0.4	1.1
chr12	7060300	CHH	0.1	0.4
chr12	7060307	CHH	0.2	0.5
chr12	7060308	CHH	0.6	0.7
chr12	7060312	CHG	0.4	0.3
chr12	7060315	CHH	0.2	0.3
chr12	7060325	CHH	0.5	0.6
chr12	7060327	CHH	0.3	0.1
chr12	7060335	CHH	0.8	0.2
chr12	7060340	CHH	0.6	0.3
chr12	7060341	CHH	0.2	1.0
chr12	7060345	CHH	0.2	0.3
chr12	7060347	CHH	0.6	0.4
chr12	7060348	CHG	0.5	0.4
chr12	7060353	CHH	0.3	0.3
chr12	7060354	CHH	0.1	0.3
chr12	7060363	CHG	0.5	0.5
chr12	7060367	CpG-12	6.1	6.4
chr12	7060382	CHH	0.3	0.7
chr12	7060383	CHH	0.7	0.5
chr12	7060384	CHG	0.1	0.4
chr12	7060385	CpG-13	22.7	16.1
chr12	7060388	CHH	0.6	0.3
chr12	7060389	CHH	0.2	0.3
chr12	7060390	CHH	0.2	0.2
chr12	7060391	CHH	1.4	0.5
chr12	7060393	CHH	0.2	0.3
chr12	7060394	CHH	0.4	0.3
chr12	7060395	CHH	0.1	0.3
chr12	7060396	CHH	0.1	0.2
chr12	7060397	CHG	1.2	0.2
chr12	7060402	CHH	0.3	0.3
chr12	7060403	CHH	0.4	0.4
chr12	7060405	CHH	0.8	0.3
chr12	7060406	CHH	0.2	0.3
chr12	7060407	CHG	0.2	0.2
chr12	7060410	CHH	0.4	1.0
chr12	7060412	CHG	0.4	0.2
chr12	7060415	CHH	0.6	0.4
chr12	7060418	CHH	0.3	0.9
chr12	7060420	CHH	0.3	0.8
chr12	7060423	CHH	0.3	0.5
chr12	7060424	CHH	0.5	0.5
chr12	7060425	CHH	0.8	0.4
chr12	7060429	CHG	0.2	0.3
chr12	7060434	CHH	0.4	0.4
chr12	7060436	CHH	0.1	0.6
chr12	7060442	CpG-14	1.9	1.7
chr12	7060451	CHH	0.1	0.2
chr12	7060461	CHH	0.1	0.3
chr12	7060462	CHH	0.4	0.3
chr12	7060463	CHH	0.1	0.7
chr12	7060464	CHH	0.0	0.4
chr12	7060465	CHH	0.0	0.5
chr12	7060473	CpG-15	1.1	1.4
chr12	7060476	CHG	0.3	0.5
chr12	7060477	CpG-16	3.6	3.1
chr12	7060479	CpG-17	6.6	6.4
chr12	7060481	CHH	0.3	0.3
chr12	7060482	CHH	0.2	0.3
chr12	7060484	CHH	0.3	0.6
chr12	7060487	CHH	0.2	0.4
chr12	7060488	CHG	0.2	0.6
chr12	7060492	CHH	0.3	0.3
chr12	7060493	CHH	0.2	0.4
chr12	7060494	CHG	0.1	0.2
chr12	7060495	CpG-18	23.5	21.0
chr12	7060497	CHH	0.5	0.3
chr12	7060498	CHH	0.3	0.5
chr12	7060499	CHG	0.1	0.2
chr12	7060502	CHG	0.2	0.4
chr12	7060503	CpG-19	6.3	6.7

Position	Context	JAK2 WT	JAK2 V617F
7060111	CpG-1	7.6	8.0
7060127	CpG-2	32.4	25.1
7060131	CpG-3	21.1	13.1
7060152	CpG-4	30.0	25.3
7060168	CpG-5	53.2	57.3
7060186	CpG-6	19.4	28.5
7060192	CHH	0.2	0.2
7060193	CHG	0.2	0.9
7060202	CHG	0.2	0.8
7060205	CpG-7	58.7	51.7
7060213	CHG	0.7	0.5
7060216	CHG	0.3	0.5
7060237	CHG	0.5	0.7
7060238	CpG-8	48.2	47.1
7060245	CHG	0.2	0.2
7060246	CpG-9	53.2	45.0
7060249	CHG	0.2	0.6
7060256	CpG-10	61.8	56.1
7060258	CHH	0.5	0.5
7060262	CpG-11	69.1	62.0
7060275	CHG	0.2	0.2
7060280	CHH	0.3	0.3
7060281	CHG	0.3	0.2
7060291	CHH	0.1	0.4
7060296	CHH	0.1	0.3
7060298	CHH	0.4	1.1
7060300	CHH	0.1	0.4
7060307	CHH	0.2	0.5
7060308	CHH	0.6	0.7
7060312	CHG	0.4	0.3
7060315	CHH	0.2	0.3
7060325	CHH	0.5	0.6
7060327	CHH	0.3	0.1
7060335	CHH	0.8	0.2
7060340	CHH	0.6	0.3
7060341	CHH	0.2	1.0
7060345	CHH	0.2	0.3
7060347	CHH	0.6	0.4
7060348	CHG	0.5	0.4
7060353	CHH	0.3	0.3
7060354	CHH	0.1	0.3
7060363	CHG	0.5	0.5
7060367	CpG-12	6.1	6.4
7060382	CHH	0.3	0.7
7060383	CHH	0.7	0.5
7060384	CHG	0.1	0.4
7060385	CpG-13	22.7	16.1
7060388	CHH	0.6	0.3
7060389	CHH	0.2	0.3
7060390	CHH	0.2	0.2
7060391	CHH	1.4	0.5
7060393	CHH	0.2	0.3
7060394	CHH	0.4	0.3
7060395	CHH	0.1	0.3
7060396	CHH	0.1	0.2
7060397	CHG	1.2	0.2
7060402	CHH	0.3	0.3
7060403	CHH	0.4	0.4
7060405	CHH	0.8	0.3
7060406	CHH	0.2	0.3
7060407	CHG	0.2	0.2
7060410	CHH	0.4	1.0
7060412	CHG	0.4	0.2
7060415	CHH	0.6	0.4
7060418	CHH	0.3	0.9
7060420	CHH	0.3	0.8
7060423	CHH	0.3	0.5
7060424	CHH	0.5	0.5
7060425	CHH	0.8	0.4
7060429	CHG	0.2	0.3
7060434	CHH	0.4	0.4
7060436	CHH	0.1	0.6
7060442	CpG-14	1.9	1.7
7060451	CHH	0.1	0.2
7060461	CHH	0.1	0.3
7060462	CHH	0.4	0.3
7060463	CHH	0.1	0.7
7060464	CHH	0.0	0.4
7060465	CHH	0.0	0.5
7060473	CpG-15	1.1	1.4
7060476	CHG	0.3	0.5
7060477	CpG-16	3.6	3.1
7060479	CpG-17	6.6	6.4
7060481	CHH	0.3	0.3
7060482	CHH	0.2	0.3
7060484	CHH	0.3	0.6
7060487	CHH	0.2	0.4
7060488	CHG	0.2	0.6
7060492	CHH	0.3	0.3
7060493	CHH	0.2	0.4
7060494	CHG	0.1	0.2
7060495	CpG-18	23.5	21.0
7060497	CHH	0.5	0.3
7060498	CHH	0.3	0.5
7060499	CHG	0.1	0.2
7060502	CHG	0.2	0.4
7060503	CpG-19	6.3	6.7
