# Supplementary material for: JAK2V617F‐dependent down regulation of SHP‐1 expression participates in the selection of myeloproliferative neoplasm cells in the presence of TGF‐β
Source: J Cell Mol Med. 2024 Oct 21;28(20):e70138. doi: 10.1111/jcmm.70138 (PMC11492149; doi:10.1111/jcmm.70138)
Supplement: Supplementary file 3 — Figure S3. [file JCMM-28-e70138-s005.pdf]

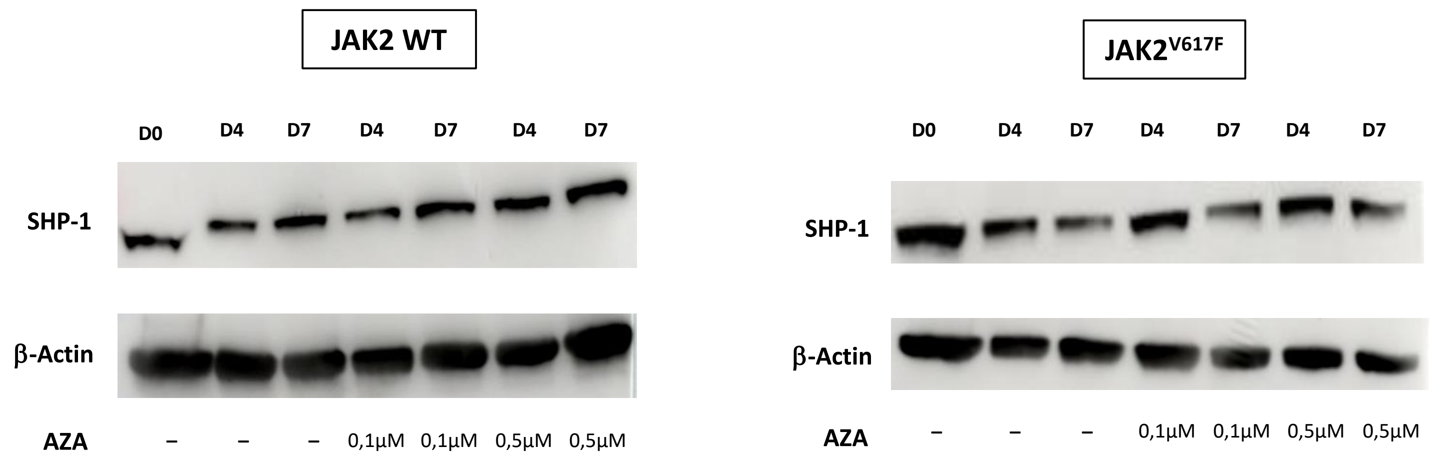

Supplementary Figure S3 : Western blot analysis of UT-7 cells expressing WT JAK2 or $JAK2^{V617F}$ after treatment with 5-AZA for 4 and 7 days.
