# Supplementary material for: JAK2V617F‐dependent down regulation of SHP‐1 expression participates in the selection of myeloproliferative neoplasm cells in the presence of TGF‐β
Source: J Cell Mol Med. 2024 Oct 21;28(20):e70138. doi: 10.1111/jcmm.70138 (PMC11492149; doi:10.1111/jcmm.70138)
Supplement: Supplementary file 4 — Figure S4. [file JCMM-28-e70138-s001.pdf]

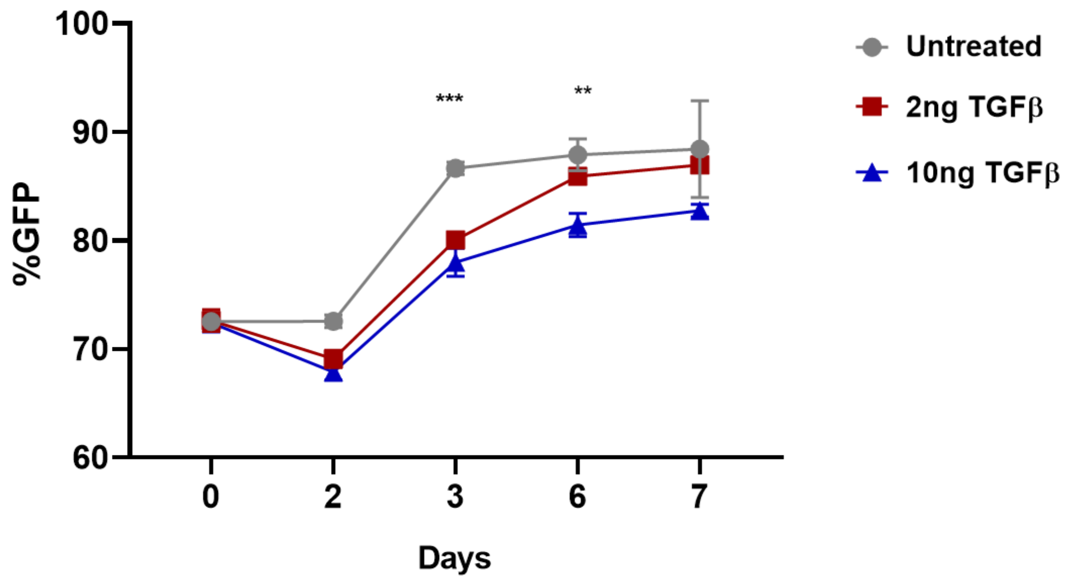

Supplementary Figure S4: Competitive experiment with a mix of JAK2^{V617F} positive UKE-1 cells transduced with either SHP-1 cDNA (70%) or empty vector (30%). The SHP-1 vector is tagged with GFP, allowing to follow the percentage of transduced cells at different days of culture with TGF-β or not. **: p<0.01 ;***: p<0.001
