# Supplementary material for: JAK2V617F‐dependent down regulation of SHP‐1 expression participates in the selection of myeloproliferative neoplasm cells in the presence of TGF‐β
Source: J Cell Mol Med. 2024 Oct 21;28(20):e70138. doi: 10.1111/jcmm.70138 (PMC11492149; doi:10.1111/jcmm.70138)
Supplement: Supplementary file 5 — Figure S5. [file JCMM-28-e70138-s003.pdf]

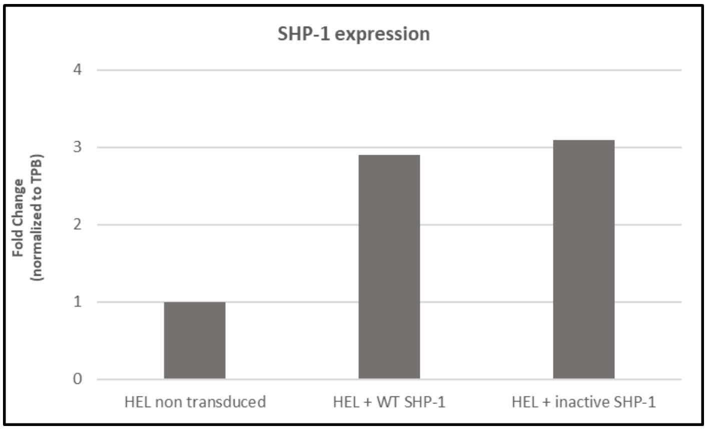


**Supplementary Figure S5** : RT-qPCR analysis of SHP-1 expression in HEL cells transduced with WT or inactive forms of SHP-1
