# Supplementary material for: JAK2V617F‐dependent down regulation of SHP‐1 expression participates in the selection of myeloproliferative neoplasm cells in the presence of TGF‐β
Source: J Cell Mol Med. 2024 Oct 21;28(20):e70138. doi: 10.1111/jcmm.70138 (PMC11492149; doi:10.1111/jcmm.70138)
Supplement: Supplementary file 6 — Table S1. [file JCMM-28-e70138-s004.pdf]

Supplementary Table 1: origin and references of the antibodies used.

Protein	Antibody
STAT-3	Cell Signaling No 9139
P-STAT3	Cell Signaling No 9145
STAT5	Cell Signaling No 94205
P-STAT5	Cell Signaling No 9351
SHP-1	Cell Signaling No 3759
SMAD4	Cell Signaling No 38454
SMAD2/3	Cell Signaling No 8685
P-SMAD2/3	R&D Systems Biotechne MAB8935
Anti-TGFβ receptor 1	Abcam ab31013
Anti-TGFβ receptor 2	Abcam ab61213
HRP-conjugated β-actin	Proteintech No HRP-60008
